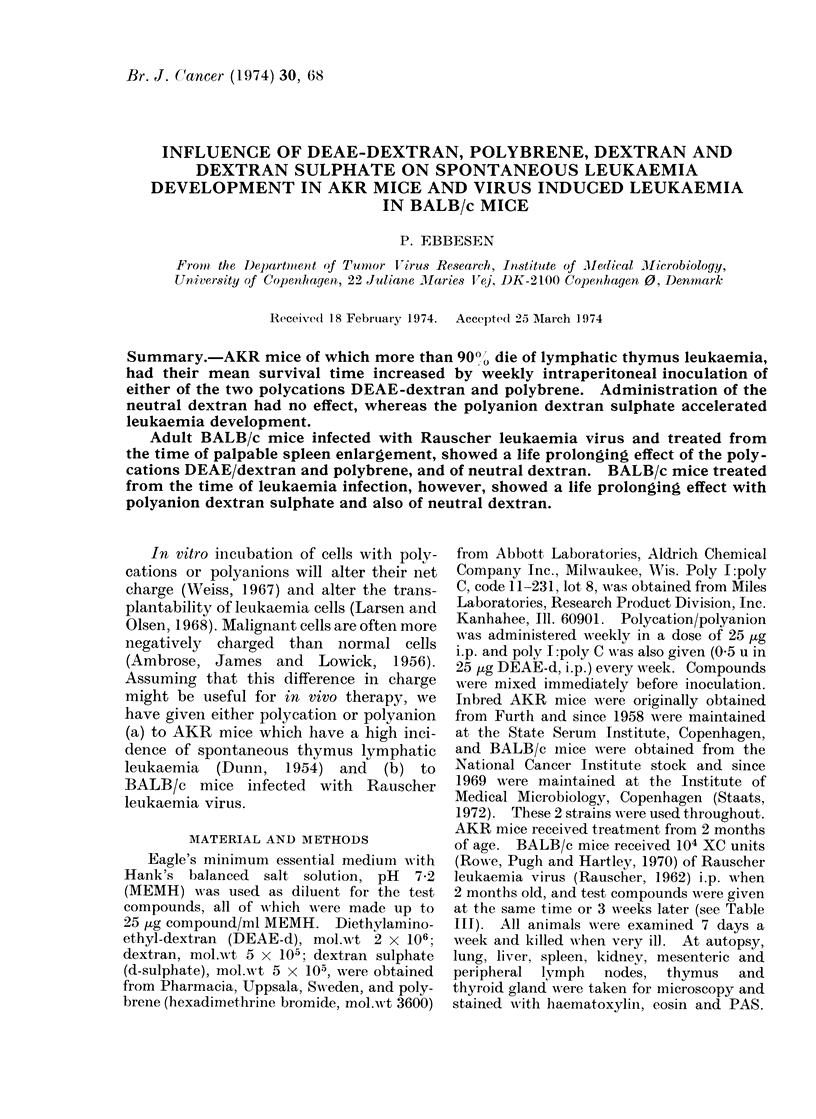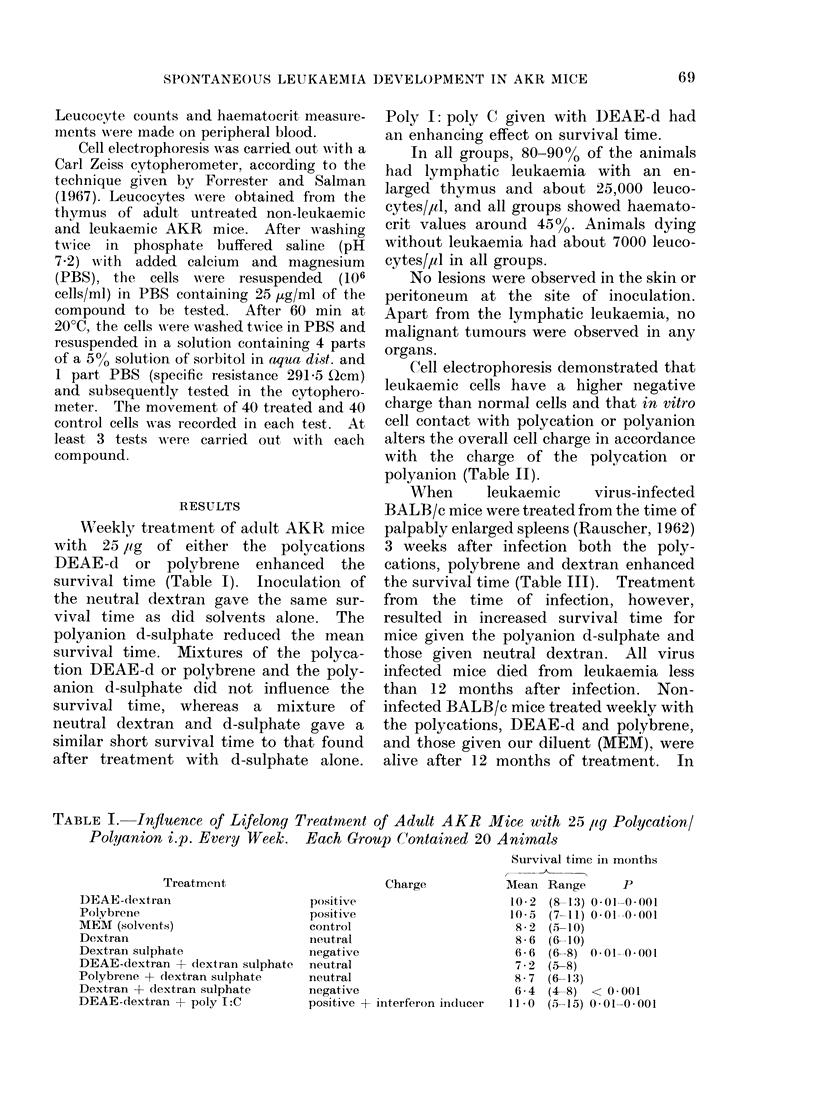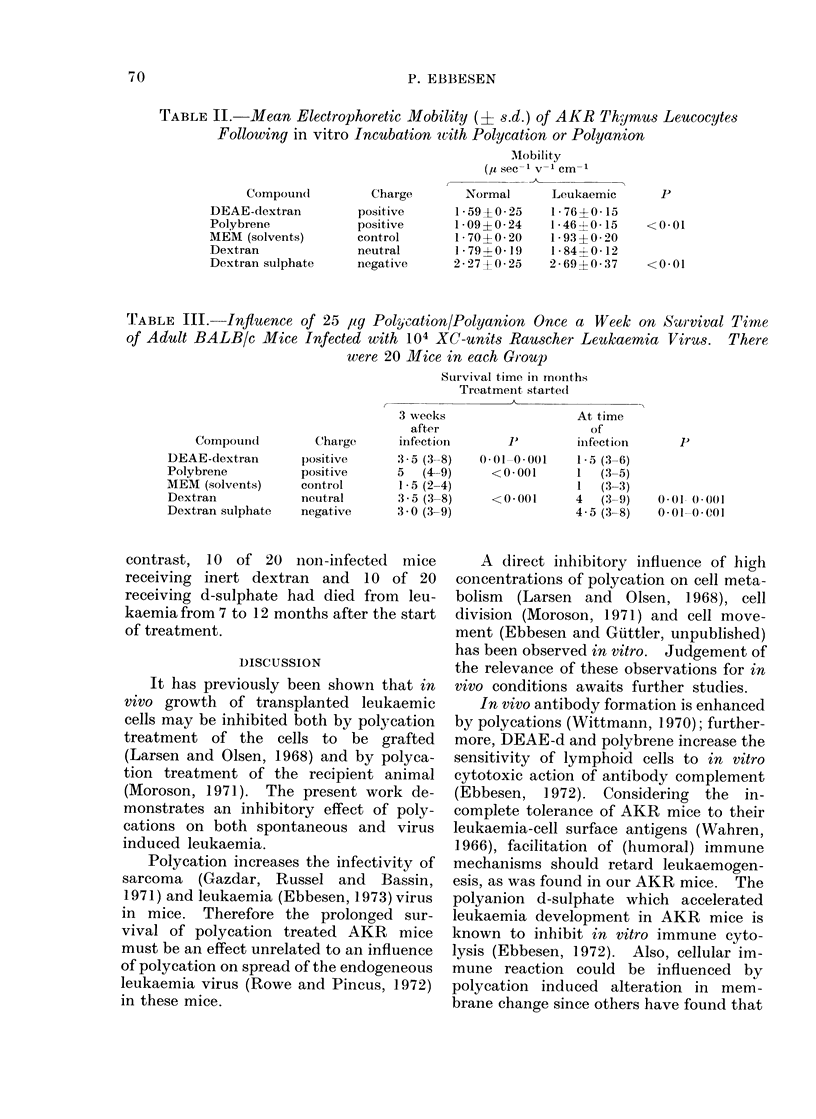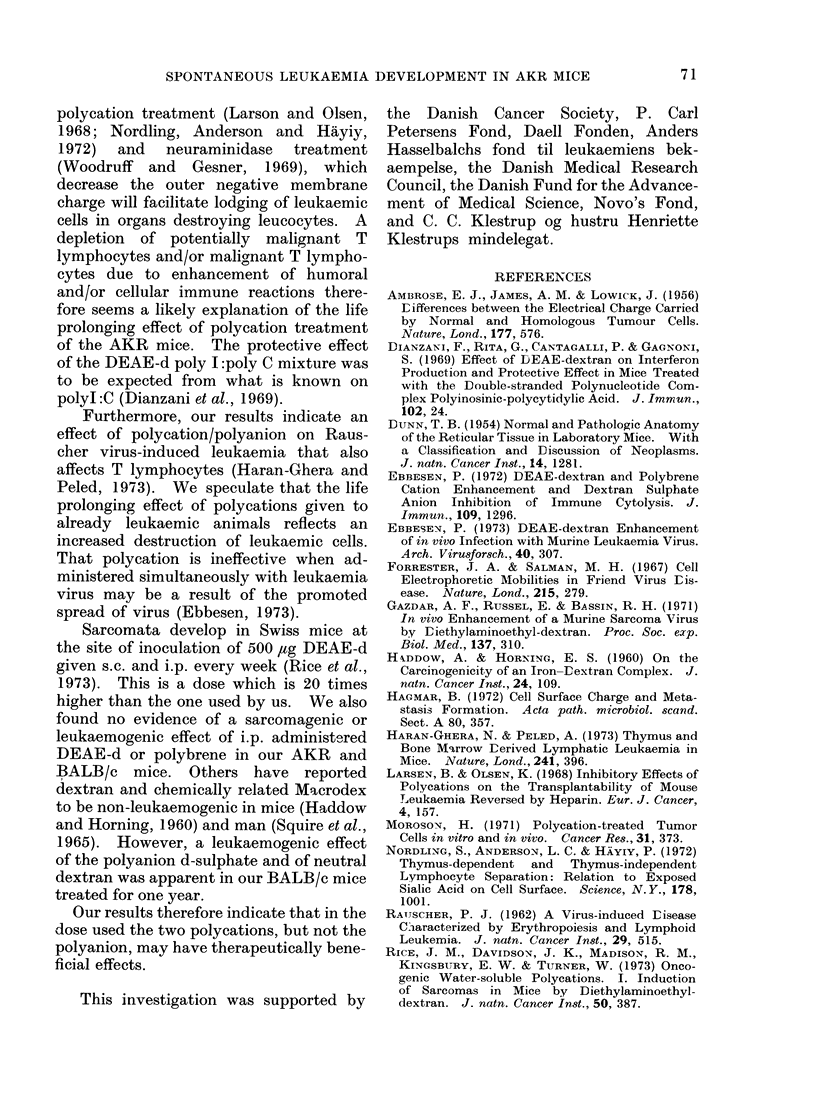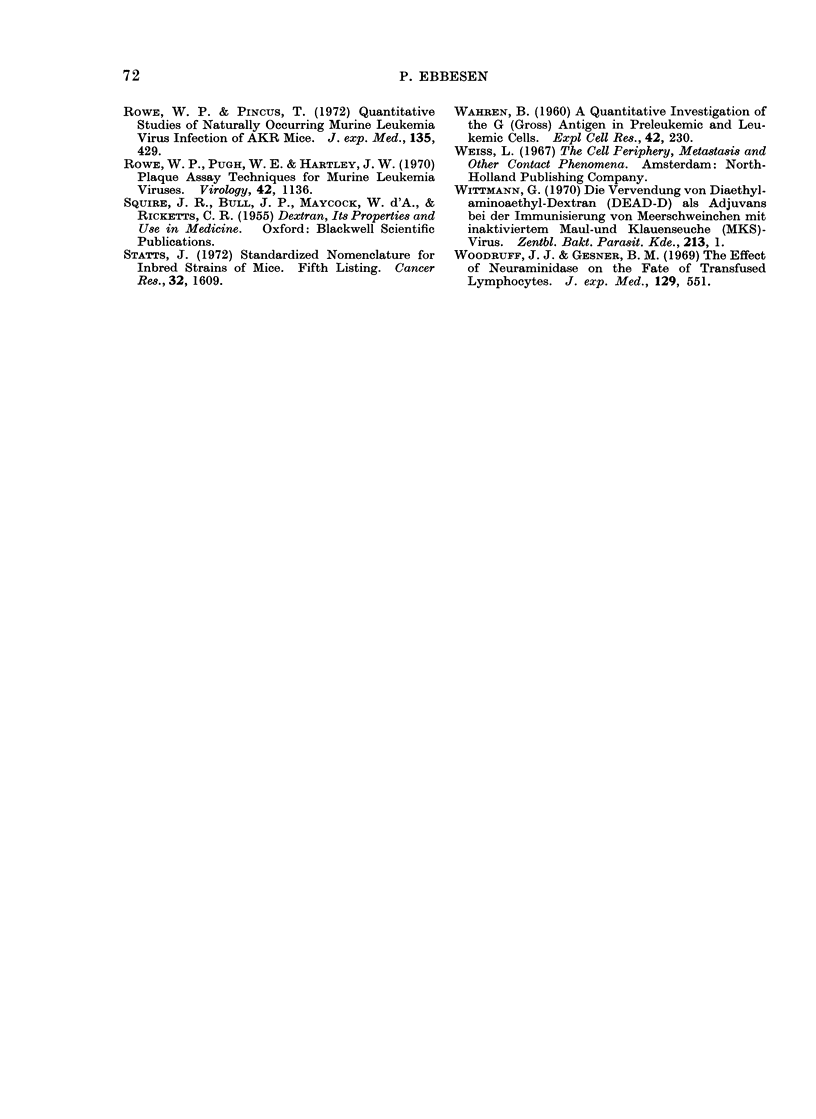# Influence of Deae-Dextran, Polybrene, Dextran and Dextran Sulphate on Spontaneous Leukaemia Development in AKR Mice and Virus Induced Leukaemia in BALB/c Mice

**DOI:** 10.1038/bjc.1974.114

**Published:** 1974-07

**Authors:** P. Ebbesen

## Abstract

AKR mice of which more than 90% die of lymphatic thymus leukaemia, had their mean survival time increased by weekly intraperitoneal inoculation of either of the two polycations DEAE-dextran and polybrene. Administration of the neutral dextran had no effect, whereas the polyanion dextran sulphate accelerated leukaemia development.

Adult BALB/c mice infected with Rauscher leukaemia virus and treated from the time of palpable spleen enlargement, showed a life prolonging effect of the polycations DEAE/dextran and polybrene, and of neutral dextran. BALB/c mice treated from the time of leukaemia infection, however, showed a life prolonging effect with polyanion dextran sulphate and also of neutral dextran.


*Br. J. Cancer* (1974) **30**, 68

### INFLUENCE OF DEAE-DEXTRAN, POLYBRENE, DEXTRAN AND DEXTRAN SULPHATE ON SPONTANEOUS LEUKAEMIA DEVELOPMENT IN AKR MICE AND VIRUS INDUCED LEUKAEMIA IN BALB/c MICE

P. EBBESEN

*From the Department of Tumor Virus Research, Institute of Medical Microbiology, University of Copenhagen, 22 Juliane Maries Vej, DK-2100 Copenhagen Ø, Denmark*

Received 18 February 1974. Accepted 25 March 1974

**Summary.—AKR mice of which more than 90% die of lymphatic thymus leukaemia, had their mean survival time increased by weekly intraperitoneal inoculation of either of the two polycations DEAE-dextran and polybrene. Administration of the neutral dextran had no effect, whereas the polyanion dextran sulphate accelerated leukaemia development.**

**Adult BALB/c mice infected with Rauscher leukaemia virus and treated from the time of palpable spleen enlargement, showed a life prolonging effect of the polycations DEAE/dextran and polybrene, and of neutral dextran. BALB/c mice treated from the time of leukaemia infection, however, showed a life prolonging effect with polyanion dextran sulphate and also of neutral dextran.**

*In vitro* incubation of cells with polycations or polyanions will alter their net charge (Weiss, 1967) and alter the transplantability of leukaemia cells (Larsen and Olsen, 1968). Malignant cells are often more negatively charged than normal cells (Ambrose, James and Lowick, 1956). Assuming that this difference in charge might be useful for *in vivo* therapy, we have given either polycation or polyanion (a) to AKR mice which have a high incidence of spontaneous thymus lymphatic leukaemia (Dunn, 1954) and (b) to BALB/c mice infected with Rauscher leukaemia virus.

### MATERIAL AND METHODS

Eagle's minimum essential medium with Hank's balanced salt solution, pH 7·2 (MEMH) was used as diluent for the test compounds, all of which were made up to 25 μg compound/ml MEMH. Diethylaminoethyl-dextran (DEAE-d), mol.wt $2 \times 10^6$; dextran, mol.wt $5 \times 10^5$; dextran sulphate (d-sulphate), mol.wt $5 \times 10^5$, were obtained from Pharmacia, Uppsala, Sweden, and polybrene (hexadimethrine bromide, mol.wt 3600) from Abbott Laboratories, Aldrich Chemical Company Inc., Milwaukee, Wis. Poly I:poly C, code 11–231, lot 8, was obtained from Miles Laboratories, Research Product Division, Inc. Kanhahee, Ill. 60901. Polycation/polyanion was administered weekly in a dose of 25 μg i.p. and poly I:poly C was also given (0·5 u in 25 μg DEAE-d, i.p.) every week. Compounds were mixed immediately before inoculation. Inbred AKR mice were originally obtained from Furth and since 1958 were maintained at the State Serum Institute, Copenhagen, and BALB/c mice were obtained from the National Cancer Institute stock and since 1969 were maintained at the Institute of Medical Microbiology, Copenhagen (Staats, 1972). These 2 strains were used throughout. AKR mice received treatment from 2 months of age. BALB/c mice received $10^4$ XC units (Rowe, Pugh and Hartley, 1970) of Rauscher leukaemia virus (Rauscher, 1962) i.p. when 2 months old, and test compounds were given at the same time or 3 weeks later (see Table III). All animals were examined 7 days a week and killed when very ill. At autopsy, lung, liver, spleen, kidney, mesenteric and peripheral lymph nodes, thymus and thyroid gland were taken for microscopy and stained with haematoxylin, eosin and PAS.

Leucocyte counts and haematocrit measurements were made on peripheral blood.

Cell electrophoresis was carried out with a Carl Zeiss cytopherometer, according to the technique given by Forrester and Salman (1967). Leucocytes were obtained from the thymus of adult untreated non-leukaemic and leukaemic AKR mice. After washing twice in phosphate buffered saline (pH 7·2) with added calcium and magnesium (PBS), the cells were resuspended ($10^6$ cells/ml) in PBS containing 25 $\mu$g/ml of the compound to be tested. After 60 min at 20°C, the cells were washed twice in PBS and resuspended in a solution containing 4 parts of a 5% solution of sorbitol in *aqua dist.* and 1 part PBS (specific resistance 291·5 Ωcm) and subsequently tested in the cytopherometer. The movement of 40 treated and 40 control cells was recorded in each test. At least 3 tests were carried out with each compound.

### RESULTS

Weekly treatment of adult AKR mice with 25 $\mu$g of either the polycations DEAE-d or polybrene enhanced the survival time (Table I). Inoculation of the neutral dextran gave the same survival time as did solvents alone. The polyanion d-sulphate reduced the mean survival time. Mixtures of the polycation DEAE-d or polybrene and the polyanion d-sulphate did not influence the survival time, whereas a mixture of neutral dextran and d-sulphate gave a similar short survival time to that found after treatment with d-sulphate alone. Poly I: poly C given with DEAE-d had an enhancing effect on survival time.

In all groups, 80–90% of the animals had lymphatic leukaemia with an enlarged thymus and about 25,000 leucocytes/$\mu$l, and all groups showed haematocrit values around 45%. Animals dying without leukaemia had about 7000 leucocytes/$\mu$l in all groups.

No lesions were observed in the skin or peritoneum at the site of inoculation. Apart from the lymphatic leukaemia, no malignant tumours were observed in any organs.

Cell electrophoresis demonstrated that leukaemic cells have a higher negative charge than normal cells and that *in vitro* cell contact with polycation or polyanion alters the overall cell charge in accordance with the charge of the polycation or polyanion (Table II).

When leukaemic virus-infected BALB/c mice were treated from the time of palpably enlarged spleens (Rauscher, 1962) 3 weeks after infection both the polycations, polybrene and dextran enhanced the survival time (Table III). Treatment from the time of infection, however, resulted in increased survival time for mice given the polyanion d-sulphate and those given neutral dextran. All virus infected mice died from leukaemia less than 12 months after infection. Non-infected BALB/c mice treated weekly with the polycations, DEAE-d and polybrene, and those given our diluent (MEM), were alive after 12 months of treatment. In

TABLE I.—*Influence of Lifelong Treatment of Adult AKR Mice with 25 $\mu$g Polycation/ Polyanion i.p. Every Week. Each Group Contained 20 Animals*

| Treatment | Charge | Survival time in months: Mean | Range | P |
|---|---|---|---|---|
| DEAE-dextran | positive | 10·2 | (8–13) | 0·01–0·001 |
| Polybrene | positive | 10·5 | (7–11) | 0·01–0·001 |
| MEM (solvents) | control | 8·2 | (5–10) | |
| Dextran | neutral | 8·6 | (6–10) | |
| Dextran sulphate | negative | 6·6 | (6–8) | 0·01–0·001 |
| DEAE-dextran + dextran sulphate | neutral | 7·2 | (5–8) | |
| Polybrene + dextran sulphate | neutral | 8·7 | (6–13) | |
| Dextran + dextran sulphate | negative | 6·4 | (4–8) | < 0·001 |
| DEAE-dextran + poly I:C | positive + interferon inducer | 11·0 | (5–15) | 0·01–0·001 |

TABLE II.—*Mean Electrophoretic Mobility* (± *s.d.*) *of AKR Thymus Leucocytes Following* in vitro *Incubation with Polycation or Polyanion*

| Compound | Charge | Mobility ($\mu$ sec$^{-1}$ v$^{-1}$ cm$^{-1}$ Normal | Leukaemic | *P* |
|---|---|---|---|---|
| DEAE-dextran | positive | 1·59±0·25 | 1·76±0·15 | |
| Polybrene | positive | 1·09±0·24 | 1·46±0·15 | <0·01 |
| MEM (solvents) | control | 1·70±0·20 | 1·93±0·20 | |
| Dextran | neutral | 1·79±0·19 | 1·84±0·12 | |
| Dextran sulphate | negative | 2·27±0·25 | 2·69±0·37 | <0·01 |

TABLE III.—*Influence of* 25 $\mu$*g Polycation/Polyanion Once a Week on Survival Time of Adult BALB/c Mice Infected with* $10^4$ *XC-units Rauscher Leukaemia Virus. There were* 20 *Mice in each Group*

| | | Survival time in months Treatment started | | | |
|---|---|---|---|---|---|
| Compound | Charge | 3 weeks after infection | *P* | At time of infection | *P* |
| DEAE-dextran | positive | 3·5 (3–8) | 0·01–0·001 | 1·5 (3–6) | |
| Polybrene | positive | 5 (4–9) | <0·001 | 1 (3–5) | |
| MEM (solvents) | control | 1·5 (2–4) | | 1 (3–3) | |
| Dextran | neutral | 3·5 (3–8) | <0·001 | 4 (3–9) | 0·01–0·001 |
| Dextran sulphate | negative | 3·0 (3–9) | | 4·5 (3–8) | 0·01–0·001 |

contrast, 10 of 20 non-infected mice receiving inert dextran and 10 of 20 receiving d-sulphate had died from leukaemia from 7 to 12 months after the start of treatment.

### DISCUSSION

It has previously been shown that *in vivo* growth of transplanted leukaemic cells may be inhibited both by polycation treatment of the cells to be grafted (Larsen and Olsen, 1968) and by polycation treatment of the recipient animal (Moroson, 1971). The present work demonstrates an inhibitory effect of polycations on both spontaneous and virus induced leukaemia.

Polycation increases the infectivity of sarcoma (Gazdar, Russel and Bassin, 1971) and leukaemia (Ebbesen, 1973) virus in mice. Therefore the prolonged survival of polycation treated AKR mice must be an effect unrelated to an influence of polycation on spread of the endogeneous leukaemia virus (Rowe and Pincus, 1972) in these mice.

A direct inhibitory influence of high concentrations of polycation on cell metabolism (Larsen and Olsen, 1968), cell division (Moroson, 1971) and cell movement (Ebbesen and Güttler, unpublished) has been observed *in vitro*. Judgement of the relevance of these observations for *in vivo* conditions awaits further studies.

*In vivo* antibody formation is enhanced by polycations (Wittmann, 1970); furthermore, DEAE-d and polybrene increase the sensitivity of lymphoid cells to *in vitro* cytotoxic action of antibody complement (Ebbesen, 1972). Considering the incomplete tolerance of AKR mice to their leukaemia-cell surface antigens (Wahren, 1966), facilitation of (humoral) immune mechanisms should retard leukaemogenesis, as was found in our AKR mice. The polyanion d-sulphate which accelerated leukaemia development in AKR mice is known to inhibit *in vitro* immune cytolysis (Ebbesen, 1972). Also, cellular immune reaction could be influenced by polycation induced alteration in membrane change since others have found that polycation treatment (Larson and Olsen, 1968; Nordling, Anderson and Häyiy, 1972) and neuraminidase treatment (Woodruff and Gesner, 1969), which decrease the outer negative membrane charge will facilitate lodging of leukaemic cells in organs destroying leucocytes. A depletion of potentially malignant T lymphocytes and/or malignant T lymphocytes due to enhancement of humoral and/or cellular immune reactions therefore seems a likely explanation of the life prolonging effect of polycation treatment of the AKR mice. The protective effect of the DEAE-d poly I:poly C mixture was to be expected from what is known on polyI:C (Dianzani *et al.*, 1969).

Furthermore, our results indicate an effect of polycation/polyanion on Rauscher virus-induced leukaemia that also affects T lymphocytes (Haran-Ghera and Peled, 1973). We speculate that the life prolonging effect of polycations given to already leukaemic animals reflects an increased destruction of leukaemic cells. That polycation is ineffective when administered simultaneously with leukaemia virus may be a result of the promoted spread of virus (Ebbesen, 1973).

Sarcomata develop in Swiss mice at the site of inoculation of 500 $\mu$g DEAE-d given s.c. and i.p. every week (Rice *et al.*, 1973). This is a dose which is 20 times higher than the one used by us. We also found no evidence of a sarcomagenic or leukaemogenic effect of i.p. administered DEAE-d or polybrene in our AKR and BALB/c mice. Others have reported dextran and chemically related Macrodex to be non-leukaemogenic in mice (Haddow and Horning, 1960) and man (Squire *et al.*, 1965). However, a leukaemogenic effect of the polyanion d-sulphate and of neutral dextran was apparent in our BALB/c mice treated for one year.

Our results therefore indicate that in the dose used the two polycations, but not the polyanion, may have therapeutically beneficial effects.

This investigation was supported by the Danish Cancer Society, P. Carl Petersens Fond, Daell Fonden, Anders Hasselbalchs fond til leukaemiens bekaempelse, the Danish Medical Research Council, the Danish Fund for the Advancement of Medical Science, Novo's Fond, and C. C. Klestrup og hustru Henriette Klestrups mindelegat.

### REFERENCES

AMBROSE, E. J., JAMES, A. M. & LOWICK, J. (1956) Differences between the Electrical Charge Carried by Normal and Homologous Tumour Cells. *Nature, Lond.*, **177**, 576.

DIANZANI, F., RITA, G., CANTAGALLI, P. & GAGNONI, S. (1969) Effect of DEAE-dextran on Interferon Production and Protective Effect in Mice Treated with the Double-stranded Polynucleotide Complex Polyinosinic-polycytidylic Acid. *J. Immun.*, **102**, 24.

DUNN, T. B. (1954) Normal and Pathologic Anatomy of the Reticular Tissue in Laboratory Mice. With a Classification and Discussion of Neoplasms. *J. natn. Cancer Inst.*, **14**, 1281.

EBBESEN, P. (1972) DEAE-dextran and Polybrene Cation Enhancement and Dextran Sulphate Anion Inhibition of Immune Cytolysis. *J. Immun.*, **109**, 1296.

EBBESEN, P. (1973) DEAE-dextran Enhancement of *in vivo* Infection with Murine Leukaemia Virus. *Arch. Virusforsch.*, **40**, 307.

FORRESTER, J. A. & SALMAN, M. H. (1967) Cell Electrophoretic Mobilities in Friend Virus Disease. *Nature, Lond.*, **215**, 279.

GAZDAR, A. F., RUSSEL, E. & BASSIN, R. H. (1971) *In vivo* Enhancement of a Murine Sarcoma Virus by Diethylaminoethyl-dextran. *Proc. Soc. exp. Biol. Med.*, **137**, 310.

HADDOW, A. & HORNING, E. S. (1960) On the Carcinogenicity of an Iron–Dextran Complex. *J. natn. Cancer Inst.*, **24**, 109.

HAGMAR, B. (1972) Cell Surface Charge and Metastasis Formation. *Acta path. microbiol. scand.* Sect. A 80, 357.

HARAN-GHERA, N. & PELED, A. (1973) Thymus and Bone Marrow Derived Lymphatic Leukaemia in Mice. *Nature, Lond.*, **241**, 396.

LARSEN, B. & OLSEN, K. (1968) Inhibitory Effects of Polycations on the Transplantability of Mouse Leukaemia Reversed by Heparin. *Eur. J. Cancer*, **4**, 157.

MOROSON, H. (1971) Polycation-treated Tumor Cells *in vitro* and *in vivo*. *Cancer Res.*, **31**, 373.

NORDLING, S., ANDERSON, L. C. & HÄYIY, P. (1972) Thymus-dependent and Thymus-independent Lymphocyte Separation: Relation to Exposed Sialic Acid on Cell Surface. *Science, N.Y.*, **178**, 1001.

RAUSCHER, P. J. (1962) A Virus-induced Disease Characterized by Erythropoiesis and Lymphoid Leukemia. *J. natn. Cancer Inst.*, **29**, 515.

RICE, J. M., DAVIDSON, J. K., MADISON, R. M., KINGSBURY, E. W. & TURNER, W. (1973) Oncogenic Water-soluble Polycations. I. Induction of Sarcomas in Mice by Diethylaminoethyl-dextran. *J. natn. Cancer Inst.*, **50**, 387.

ROWE, W. P. & PINCUS, T. (1972) Quantitative Studies of Naturally Occurring Murine Leukemia Virus Infection of AKR Mice. *J. exp. Med.*, **135**, 429.

ROWE, W. P., PUGH, W. E. & HARTLEY, J. W. (1970) Plaque Assay Techniques for Murine Leukemia Viruses. *Virology*, **42**, 1136.

SQUIRE, J. R., BULL, J. P., MAYCOCK, W. d'A., & RICKETTS, C. R. (1955) *Dextran, Its Properties and Use in Medicine.* Oxford: Blackwell Scientific Publications.

STATTS, J. (1972) Standardized Nomenclature for Inbred Strains of Mice. Fifth Listing. *Cancer Res.*, **32**, 1609.

WAHREN, B. (1960) A Quantitative Investigation of the G (Gross) Antigen in Preleukemic and Leukemic Cells. *Expl Cell Res.*, **42**, 230.

WEISS, L. (1967) *The Cell Periphery, Metastasis and Other Contact Phenomena.* Amsterdam: North-Holland Publishing Company.

WITTMANN, G. (1970) Die Vervendung von Diaethylaminoaethyl-Dextran (DEAD-D) als Adjuvans bei der Immunisierung von Meerschweinchen mit inaktiviertem Maul-und Klauenseuche (MKS)-Virus. *Zentbl. Bakt. Parasit. Kde.*, **213**, 1.

WOODRUFF, J. J. & GESNER, B. M. (1969) The Effect of Neuraminidase on the Fate of Transfused Lymphocytes. *J. exp. Med.*, **129**, 551.